# Metabolic strategy of macrophages under homeostasis or immune stress in *Drosophila*

**DOI:** 10.1007/s42995-022-00134-1

**Published:** 2022-08-16

**Authors:** Wang Luo, Sumin Liu, Fang Zhang, Long Zhao, Ying Su

**Affiliations:** 1 Institute of Evolution and Marine Biodiversity, Ocean University of China, Qingdao, 266003 China; 2 College of Marine Life Sciences, Ocean University of China, Qingdao, 266003 China; 3 Fisheries College, Ocean University of China, Qingdao, 266003 China; 4 Key Laboratory of Mariculture (OUC), Ministry of Education, Qingdao, 266003 China

**Keywords:** Macrophage, *Drosophila*, Immune system, Metabolism, Plasmatocyte

## Abstract

Macrophages are well known for their phagocytic functions in innate immunity across species. In mammals, they rapidly consume a large amount of energy by shifting their metabolism from mitochondrial oxidative phosphorylation toward aerobic glycolysis, to perform the effective bactericidal function upon infection. Meanwhile, they strive for sufficient energy resources by restricting systemic metabolism. In contrast, under nutrient deprivation, the macrophage population is down-regulated to save energy for survival. *Drosophila melanogaster* possesses a highly conserved and comparatively simple innate immune system. Intriguingly, recent studies have shown that *Drosophila* plasmatocytes, the macrophage-like blood cells, adopt comparable metabolic remodeling and signaling pathways to achieve energy reassignment when challenged by pathogens, indicating the conservation of such metabolic strategies between insects and mammals. Here, focusing on *Drosophila* macrophages (plasmatocytes), we review recent advances regarding their comprehensive roles in local or systemic metabolism under homeostasis or stress, emphasizing macrophages as critical players in the crosstalk between the immune system and organic metabolism from a *Drosophila* perspective.

## Introduction

The immune system in *Drosophila* comprises two major arms, the humoral immune response and the cellular immune response, in response to bacterial and fungal infections (Bajgar et al. 2021; Lu et al. 2020). The humoral response includes the induction of antimicrobial peptides (AMPs) via the Toll and IMD (immune deficiency) signaling pathways (Yi et al. 2014). The fat body, a functional homolog of the vertebrate liver, is the primary tissue producing AMPs and secreting them into the body cavity in response to a stimulus (Lemaitre and Hoffmann 2007). The cellular response in *Drosophila* is performed through two primary mechanisms, i.e., phagocytosis and encapsulation, by circulating blood cells (hemocytes) in the body cavity (Lemaitre and Hoffmann 2007).

In *Drosophila*, at least three types of blood cells akin to vertebrate myeloid cells have been identified: plasmatocytes, crystal cells, and lamellocytes (Evans et al. 2003; Lan et al. 2020; Mukherjee et al. 2011; P et al. 2020). Plasmatocytes, representing 90–95% of all mature hemocytes, are phagocytes and are very similar to cells of the mammalian innate immune system, in particular macrophages and neutrophils (Bajgar et al. 2021). Crystal cells, which constitute ~ 5% of mature hemocytes, are non-phagocytic but express the oxidoreductase prophenoloxidase (proPO) to mediate the melanization process (Bidla et al. 2007; Mukherjee et al. 2011). Lamellocytes are large, flat cells that primarily function in encapsulating objects too large to be phagocytosed. Unlike plasmatocytes and crystal cells, lamellocytes are not found in embryos or adults, but are only produced in larvae. Furthermore, they are rarely observed in healthy larvae but are rapidly and massively produced upon parasitic wasp infection (Evans et al. 2003; Rizki and Rizki 1992).

These blood cells are sentinels in host defense, especially the macrophage-like plasmatocytes, the phagocytic function of which is highly conserved in the course of the evolution (Bajgar et al. 2021). These immune cells require a large amount of energy to perform their bactericidal function in the critical moment of bacterial infection. In mammals, activated immune cells, such as monocytes, macrophages, and neutrophils, employ aerobic glycolysis to satisfy rapid energy demands (Pearce and Pearce 2013), and accumulate immuno-metabolites to facilitate the antimicrobial response (Rosenberg et al. 2022). Similarly, the *Drosophila* macrophage-like plasmatocytes adopt comparable metabolic strategies and signaling pathways when challenged by pathogens (Bajgar and Dolezal 2018; Krejcova et al. 2019). In addition, they strive for sufficient energy resources from systemic development and metabolism to supply their immune behaviors (Bajgar et al. 2021). Here, we review the recent advances regarding the comprehensive roles of macrophages in local or systemic metabolism. Since the involved processes in *Drosophila* and mammals are highly conserved during evolution, the knowledge gained from either *Drosophila* or mammalian studies would both provide significant insights for fully understanding the metabolic adaptability in immune systems.

## *Drosophila* hematopoiesis

**Fig. 1:**
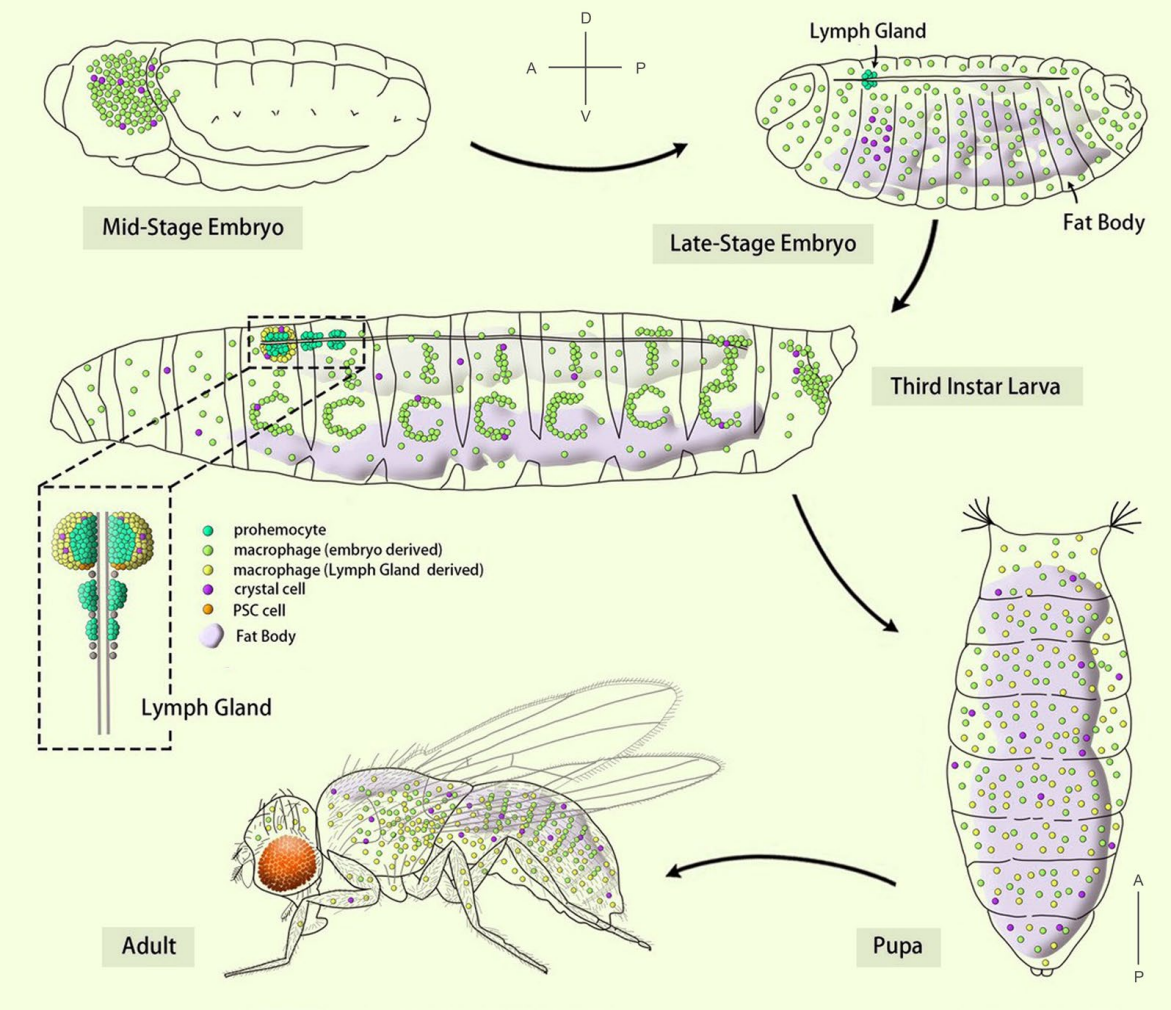
*Drosophila* hematopoiesis. The anterior (A)-posterior (P) and dorsal (D)-ventral (V) axes indicate the orientation of embryo/larva/adult, except the pupa is orientated with anterior to the upside and posterior to the downside. *PSC* posterior signaling center

*Drosophila melanogaster* is a well-established animal model employed to study the hematopoietic processes, through which the immune cells are generated. Like in vertebrates, *Drosophila* hematopoiesis occurs in two spatially and temporally distinct waves (Fig. 1) (Crozatier and Meister 2007; Evans et al. 2003; Rodrigues et al. 2021). The first wave of *Drosophila* hematopoiesis occurs during embryogenesis in the head mesoderm and gives rise to approximately 700 plasmatocytes and 30 crystal cells (Tepass et al. 1994; Wood and Jacinto 2007). These embryonically derived blood cells comprise the larval circulatory hemocytes and the sessile pools of blood cells in subcuticular hematopoietic pockets (Holz et al. 2003; Wood and Jacinto 2007). The second wave occurs in the larval stage. In addition to the proliferation of the sessile blood cells in subcuticular hematopoietic pockets, the lymph gland, a specialized hematopoietic organ, contributes mainly to the blood cell population (Makhijani et al. 2011; Morin-Poulard et al. 2021). In the lymph gland, the blood progenitor cells differentiate into mature blood cells, including plasmatocytes, crystal cells, and lamellocytes. At the onset of metamorphosis, the lymph gland is disassociated and the blood cells within it are dispersed into the circulatory system (Grigorian et al. 2011).

*Drosophila* hematopoietic processes have developmental and functional affinities in mammals (Gold and Bruckner 2014). For instance, in humans, RUNX1 is a well-described target of chromosomal translocations leading to acute myeloid leukemia (AML) (Banerjee et al. 2019; Okuda et al. 1996; Speck and Gilliland 2002). Interestingly, the *Drosophila* ortholog of RUNX1, Lozenge (Lz), is an essential transcription factor for crystal cell specification and development during both waves of hematopoiesis (Bras et al. 2012; Dyer et al. 2017; Lebestky et al. 2000; Miller et al. 2017). Myeloid leukemia factor 1 (MLF1) is another identified target associated with AML (Yoneda-Kato et al. 1996). In flies, MLF is also known to control blood cell development by stabilizing Lz (Miller et al. 2017). Since the hemopoietic system in *Drosophila* is simpler than that of mammals, its study is a highly efficient way to increase knowledge and understanding of the underlying mechanisms and etiologies of human blood disease.

## Macrophages in *Drosophila*

Macrophages are present in all animals and play diverse roles serving as the front line of protection against invading pathogens (Lim et al. 2017). The most classic role of macrophages is phagocytosis (Franken et al. 2016). In vertebrates, macrophages are known to polarize into bactericidal (M1) or healing (M2) functional phenotypes in response to an activating stimulus (Mills et al. 2000). M1 macrophages are associated with tissue proinflammatory response and microbial killing. M2 macrophages are associated with the resolution of inflammation, wound healing, and are also observed under the conditions of helminth infections and allergies (Breda et al. 2019).

In *Drosophila*, plasmatocytes are macrophage-like blood cells that are differentiated from the hematopoietic precursors, prohemocytes. The GATA protein Serpent (Srp) is an early marker defining hemocyte precursors in both embryos and larvae (Jung et al. 2005; Rehorn et al. 1996). GATA family proteins are evolutionarily highly conserved transcription factors first identified in hematopoietic and cardiac development (Tremblay et al. 2018). Srp regulates the expression of *glide/glial cells missing* (*gcm*) and *gcm2*, which are critical lineage-specific transcription factors that direct the specification and terminal differentiation of embryonic plasmatocytes (Alfonso and Jones 2002; Bernardoni et al. 1997). Their effects on larval hematopoiesis, however, remain equivocal because neither is expressed in the larval lymph gland or in circulating hemocytes (Avet-Rochex et al. 2010; Bataille et al. 2005; Lebestky et al. 2000).

**Fig. 2:**
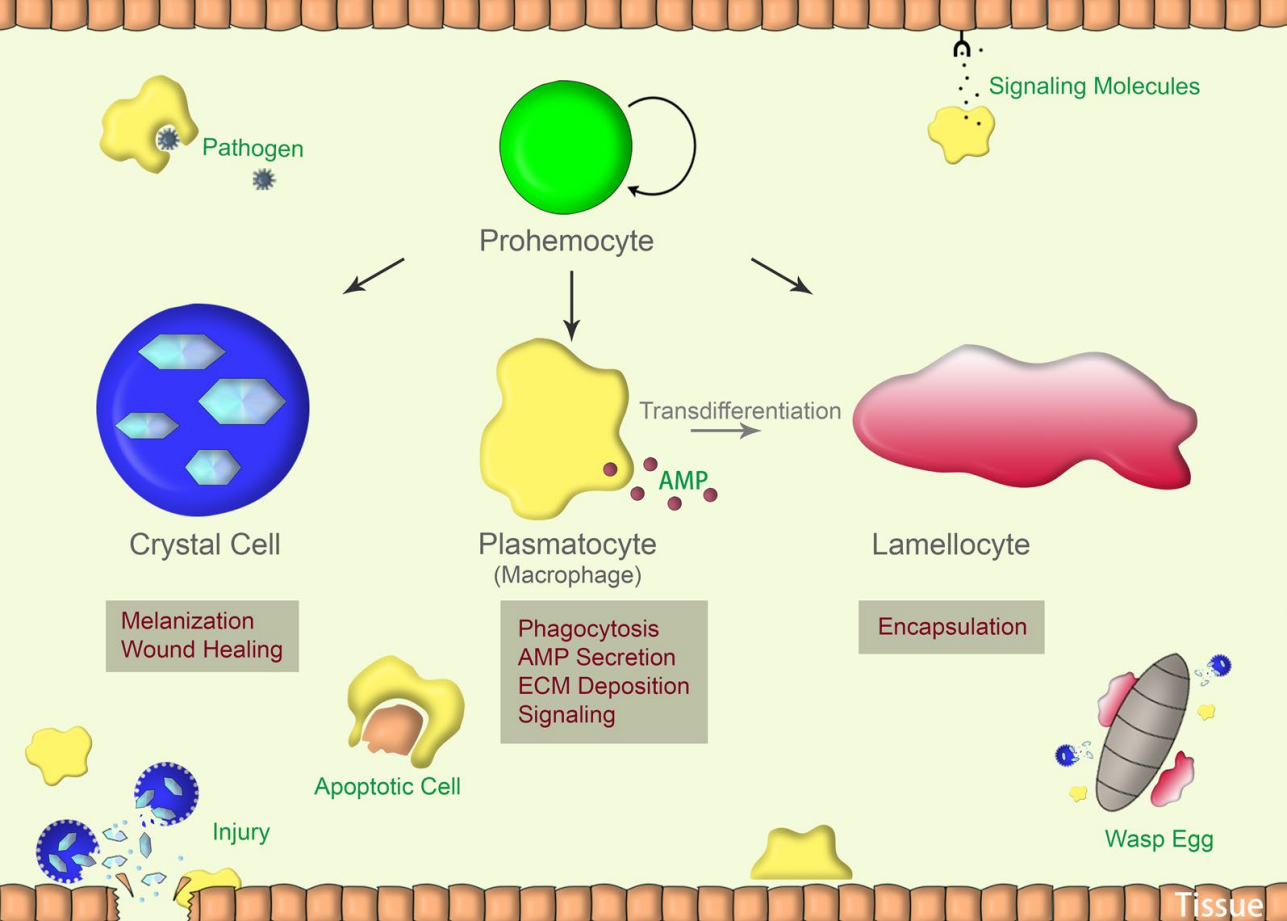
*Drosophila* hemocytes and their functions. *ECM* extracellular matrix, *AMP* antimicrobial peptides

Plasmatocytes constitute the largest population of hemocytes in *Drosophila* and show significant functional diversity (Fig. 2). Studies in the last two decades have revealed parallel multi-functions of *Drosophila* plasmatocytes, as counterparts of macrophages in mammals. In *Drosophila*, beyond many developmental functions, plasmatocytes play diverse pivotal roles during the innate immune response, including maintenance of tissue or organ homeostasis, monitoring tissue damage, recognition of apoptotic cells and, most importantly, defense against invading pathogens (Babcock et al. 2008; Bunt et al. 2010; Franc et al. 1996; Goto et al. 2003; Kierdorf et al. 2020; Mase et al. 2021; Olofsson and Page 2005; Wood and Martin 2017). In addition, plasmatocytes have the potential to trans-differentiate into lamellocytes in response to parasitic infection (Stofanko et al. 2010).

In mammals, macrophages are an extremely heterogeneous population corresponding to their diverse roles (Gordon and Pluddemann 2017). *Drosophila* plasmatocytes have long been regarded as a homogeneous population. However, multiple studies using single-cell RNA sequencing have recently revealed their heterogeneity (Cattenoz et al. 2020, 2021; Coates et al. 2021; Hartenstein 2020; Mase et al. 2021; Tattikota et al. 2020). In particular, Shin et al. (2020) reported distinctive subpopulations of plasmatocytes in embryonically derived circulating and sessile hemocytes at the larval stage, based on the expression of Peroxidasin (Pxn) and Hemolectin (Hml), two commonly used markers for plasmatocytes. Although the expression of these two markers are mostly overlap in these blood cells, subpopulations of plasmatocytes with only Hml or Pxn expression are also observed. These subtypes of plasmatocytes could be specialized to function in the different aspects of homeostasis, including immunity and metabolism. Interestingly, when Hml-positive (Hml +) hemocytes are killed upon the expression of pro-apoptotic genes, the number of remaining hemocytes (Pxn-positive and Hml-negative [Pxn + Hml-]) is dramatically increased, suggesting the existence of an intrinsic mechanism to maintain a constant population of plasmatocytes during development.

## Metabolism in macrophages shifts toward aerobic glycolysis upon infection

In vertebrates, the polarized M1 macrophages undergo an overall change in morphology and utilize aerobic glycolysis—a metabolic pathway of generating adenosine triphosphate (ATP) usually in low-oxygen environments—to quickly provide adequate energy for phagocytosis (Galvan-Pena and O’Neill 2014; O’Neill and Pearce 2016; Pearce and Pearce 2013; Van den Bossche et al. 2017). Aerobic glycolysis takes place in the cytosol and converts glucose into lactate along with the production of 2 ATPs per glucose molecule. This energy yield is much lower than that of the oxidative phosphorylation pathway, which can generate 32 ATPs from the complete oxidation of glucose through the mitochondrial tricarboxylic acid (TCA) cycle (Vaupel and Multhoff 2021). Aerobic glycolysis, however, runs much faster than oxidative phosphorylation due to having far fewer reaction steps (Chandel et al. 2021). Therefore, activated macrophages shift their metabolism toward aerobic glycolysis to meet the sharp demand for energy.

**Fig. 3:**
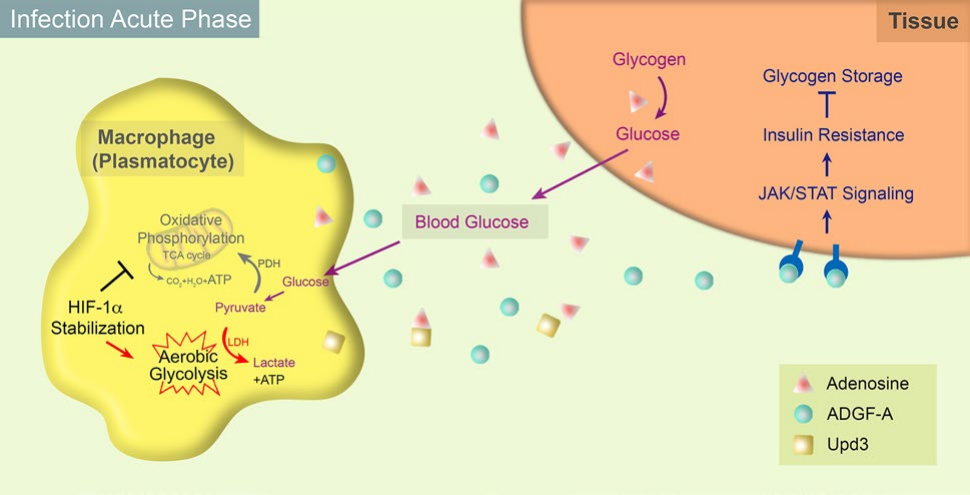
Macrophages alter local and systemic metabolisms upon infection in *Drosophila*. Upon infection, HIF-1α stabilization in activated macrophages locally promotes the metabolic switch toward the aerobic glycolysis for rapid energy production to support the effective phagocytosis. Pyruvate, the key metabolite of glucose, is generally transported into mitochondria and is converted into acetyl-CoA by pyruvate dehydrogenase (PDH) for participating in the TCA cycle. In the acute phase of infection, the mitochondrial transport of pyruvate and the following ATP production by oxidative phosphorylation are repressed. Instead, cytosolic pyruvate is converted into lactate by lactate dehydrogenase (LDH) coupled with high-speed ATP production. At the same time, the activated macrophages restrict systemic metabolisms to grab more nutrients by secreting multiple effectors. The extracellular adenosine released by macrophages is recognized or taken up by other tissues, promoting glucose production from glycogen stores and glucose release into the hemolymph. This process is negatively controlled by adenosine deaminase-related growth factor A (ADGF-A), which is also produced by macrophages and catalyzes the degradation of adenosine. As another important effector secreted from macrophages, Upd3/IL-6 amplifies JAK/STAT signaling in other tissues reducing their insulin sensitivity and glycogen storage

Like in mammalian activated (M1) macrophages, a recent study indicates that *Drosophila* macrophages (plasmatocytes) also undertake a dramatic shift toward aerobic glycolysis upon infection by pathogens (Krejcova et al. 2019). Such an evolutionary conserved metabolic reprogramming in macrophages is mediated by hypoxia-inducible factor 1 alpha (HIF-1α, known as Sima in *Drosophila*) (Corcoran and O'Neill 2016; Krejcova et al. 2019) (Fig. 3; Table 1). It is well-known that HIF-1α functions as a major regulator in response to hypoxia. HIF-1α is unstable and quickly degrades in normoxia, while in hypoxia HIF-1α becomes stabilized and is translocated into nuclei where it functions as a transcription factor to activate target genes including a set of aerobic glycolysis genes (Centanin et al. 2005; Iommarini et al. 2017). During the acute phase of infection, HIF-1α activity is increased in *Drosophila* macrophages (Krejcova et al. 2019). Previous studies have reported that nuclear factor kappa B (NF-ĸB) facilitates the stabilization of HIF-1α under normal oxygen levels in *Drosophila* and mice (Jung et al. 2003; van Uden et al. 2011). It is speculated that infection-induced NF-ĸB signaling promotes the normoxic stabilization of HIF-1α in activated macrophages (Dolezal et al. 2019). Increased HIF-1α levels enhance the transcription of a series of glycolytic genes. In particular, as a target of HIF-1α, lactate dehydrogenase (LDH) activity is elevated in macrophages upon infection to catalyze the conversion of pyruvate into lactate (Firth et al. 1995; Krejcova et al. 2019). In contrast, knocking down HIF-1α decreases the expression of aerobic glycolysis genes in activated macrophages, which leads to the metabolic preference of oxidative phosphorylation and the failure to resist infection (Krejcova et al. 2019). Therefore, HIF-1α stabilization upon infection is a crucial event to elicit the metabolic switch toward aerobic glycolysis for rapid energy production to support effective phagocytosis.

**Table 1:** List of key factors influencing metabolism directly or indirectly in *Drosophila*

Factors	Type of molecules	Sources	Targets	Functions	References
HIF-1α	Transcription regulator	Macrophages	Macrophages	Converts the metabolic strategy to aerobic glycolysis	Krejčová et al. (2019)
Adenosine	Nucleotide	Macrophages	Organism	Reflects the nutritional demands and regulates the systemic metabolism	Bajgar and Dolezal (2018); Bajgar et al. (2015)
ADGF-A	Extracellular deaminase	Macrophages	Organism	Catalyzes the degradation of adenosine	Bajgar and Dolezal (2018);
					Dolezal et al. (2005)
Upd3	Cytokine	Macrophages	Organism	Causes systemic insulin resistance	Woodcock et al. (2015)
Edin	Secreted peptide	Fat Body	Macrophages	Up-regulates sessile macrophage population and promotes their release into the circulatory system	Vanha-Aho et al. (2015)
NimB5	Adipokine	Fat Body	Macrophages	Reduces the proliferation of macrophages upon nutrient scarcity	Ramond et al. (2020)
Pvf3	PDGF-family growth factor	Macrophages	Fat body	Contributes to lipid storage	Cox et al. (2021)
Pvr	PDGF/VEGF receptor	Fat Body	Fat body	Contributes to lipid storage	Cox et al. (2021)

Another significant issue of this metabolic switch is the remodeling of mitochondrial metabolism since the TCA cycle is inactivated (Nagao et al. 2019). In M1 macrophage mitochondria, the interruption in the TCA cycle causes the accumulation of metabolic intermediates, including succinate, which attenuates the degradation of HIF-1α by prolyl hydroxylases, leading to the stabilization of HIF-1 α (Tannahill et al. 2013). In addition, mitochondria undergo continuous fission and fusion to dynamically control mitochondrial quality and function (Ray et al. 2021). The M1 macrophage is characterized by mitochondrial fission, whereas M2 macrophages that utilize oxidative phosphorylation to generate ATP have elongated mitochondria (Breda et al. 2019). In *Drosophila*, mitochondrial fission–fusion events contribute to blood progenitor differentiation and the eventual blood cell count (Ray et al. 2021). However, it is unclear whether *Drosophila* macrophage activation upon infection is also coupled with the dynamic changes of mitochondrial morphology.

## Alteration of systemic metabolism by macrophages upon infection

Macrophages lack significant nutritional storage, which means they cannot sustain persistent activation (Bajgar et al. 2021). To meet the sudden nutritional requirements during the effective immune response, the innate immune system not only converts metabolic processes in activated macrophages for rapid energy production, but also seizes nutrients as fuel from non-immune systems (Bajgar et al. 2015) (Fig. 3).

Upon infection, the activated macrophages release multiple types of signaling molecules, including adenosine, to increase nutrient uptake thereby supporting their enhanced energy requirement (Bajgar and Dolezal 2018; Bajgar et al. 2015). As an intracellular purine metabolite, an elevated adenosine concentration is typically a consequence of cellular metabolic status, such as the increased consumption of ATP (Eltzschig 2013). Upon infection by wasp eggs or bacteria, activated blood cells release adenosine into the extracellular compartment. Through adenosine receptors or transporters, the adenosine signal received in non-immune systems, such as the brain, imaginal discs, and fat bodies, inhibits their metabolic activities to save nutrients for immune cells. In the main storage organ fat body, the extracellular adenosine inhibits glycogen synthesis through AMPK, promotes the production of glucose from glycogen stores and thereby facilitates the release of glucose into the hemolymph (Aymerich et al. 2006; Bajgar and Dolezal 2018; Zuberova et al. 2010). The extracellular adenosine also suppresses the growth and development processes in larvae to free up energy for the immune response, whereas the mutant larvae that lack the adenosine receptor develop normally but fail to effectively combat the invasion (Bajgar and Dolezal 2018; Bajgar et al. 2015).

Previous studies have shown that systemic insulin resistance (IR) is able to facilitate the over-production of macrophages, a phenomenon that is conserved from *Drosophila* to humans (Straub 2014; Woodcock et al. 2015). Insulin is the key player to let blood glucose into cells for consumption to produce energy. In situations of long-term high blood glucose (hyperglycemia), cells may stop responding to insulin, a condition known as insulin resistance. Macrophages present a constitutive expression of JAK/STAT-activating cytokine Unpaired 3 (Upd3) in *Drosophila* or interleukin-6 (IL-6) in humans (Kierdorf et al. 2020), which have been considered as the critical factor inducing insulin resistance. JAK/STAT is an evolutionarily conserved signaling pathway as a critical regulator in the immune system and in a wide range of developmental and metabolic processes (Arbouzova and Zeidler 2006; Dodington et al. 2018; Ferguson and Martinez-Agosto 2017; Villarino et al. 2017). Upon infection, an increased amount of Upd3/IL-6 secreted from the activated macrophage population amplifies JAK/STAT signaling in muscles, the main reservoir for stored glycogen, reducing their insulin sensitivity and thereby suppressing their blood glucose utilization (Mashili et al. 2013; Yang and Hultmark 2017; Yang et al. 2015). Consequently, systemic insulin resistance increases the circulating glucose thus enabling the cells of the immune system to produce more energy.

## Dynamic control of metabolism in macrophages during infection

After the acute phase of the immune response, the rapid amplification of macrophages is not needed. Therefore the energy balance between the immune and non-immune systems must be re-built, otherwise the persistent high-energy cost may adversely affect the survival of the organism (Demas 2004). In addition, the nutrients reallocated to the immune cells could be utilized by the pathogens to cause worsening of the infection (Bajgar and Dolezal 2018; Passalacqua et al. 2016). For example, *Listeria monocytogenes*, a facultative intracellular bacterial pathogen found in the cytosol of *Drosophila* phagocytic cells, acquires nutrients from the host cell to support its growth and replication (Mansfield et al. 2003; Passalacqua et al. 2016). Likewise, energy for antibacterial macrophages may directly nourish the pathogens, through which the host survival is decreased (Bajgar and Dolezal 2018). Thus, organisms require proper regulation for energy allocation to avoid excessive consumption of energy.

Indeed, when the infection is cleared, the metabolic strategies adopted by activated macrophages can be reversed, for example the termination of aerobic glycolysis (Krejcova et al. 2019). During the resolution phase of infection, the immune system has killed the majority of the pathogens, the bacterial residues are cleared, and homeostasis is re-established in the host. During this period, macrophages become quiescent again, switching back to basal metabolism and prepare to re-switch their metabolic processes when faced with the next challenge by a pathogen (Mosser and Edwards 2008; Pearce and Pearce 2013). Besides the autonomous metabolic switch, the systemic metabolic alteration upon infection is negatively controlled by adenosine deaminase-related growth factor A (ADGF-A) (Bajgar and Dolezal 2018). As an adenosine-degrading enzyme, ADGF-A is expressed during the late-stage of infection and catalyzes the irreversible deamination of adenosine to inosine (Dolezal et al. 2005). Ablating ADGF-A expression in macrophages prevents degradation of adenosine, leading to prolonged energy reallocation to the immune system, glycogen storage reduction, and sometimes increased amounts of intracellular pathogens due to the availability of excessive energy (Bajgar and Dolezal 2018).

## Metabolic crosstalk between macrophages and systemic tissues

**Fig. 4:**
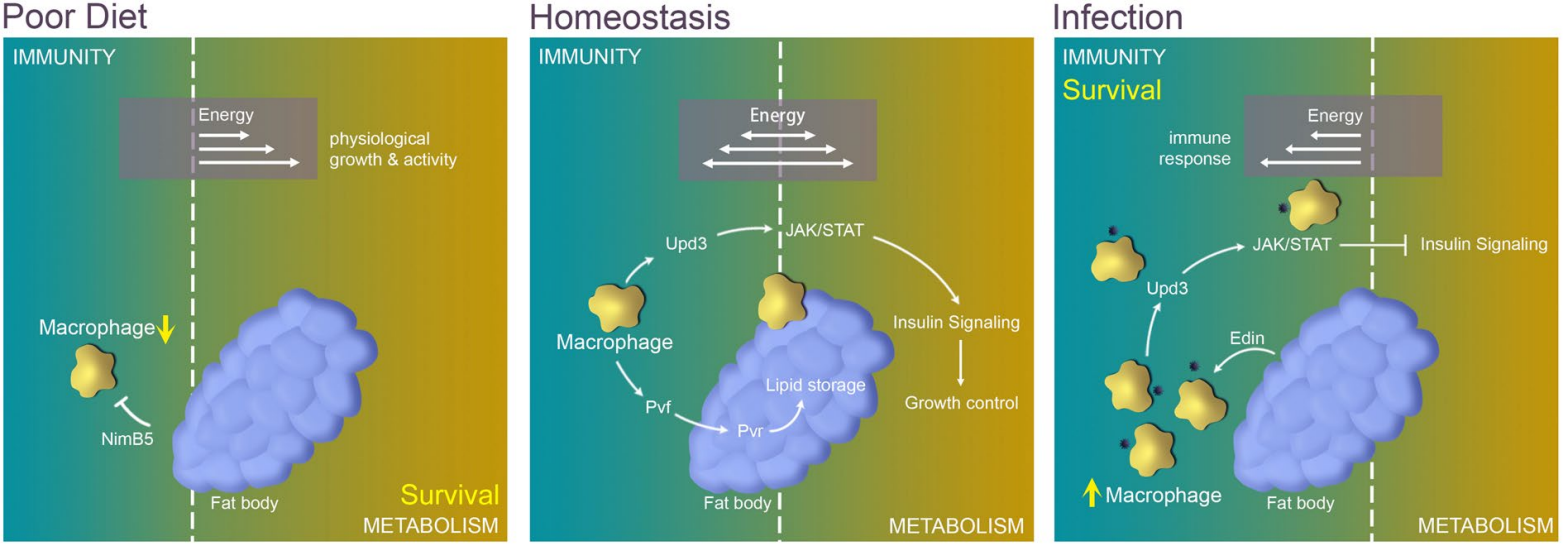
Macrophage-associated interplay between metabolism and the immune system in *Drosophila*. During homeostasis, macrophages are maintained in a basal number and at a basal metabolic level, playing essential roles to regulate lipid storage and organism growth through Pvf/Pvr signaling and JAK/STAT signaling. However, under stress conditions due to infection or nutrition deficiency, the immune system and other tissues compete for energy metabolism. Upon infection, the energy is reassigned to the immune system by inhibiting systemic metabolisms, which supports the rapid amplification of the macrophage population to defend against the pathogens. Likewise, when the nutrition is limited, the energy is reallocated toward the metabolic processes to ensure the survivability of the organism, and the number of macrophages is suppressed to save nutrients

Immunity and metabolism are intimately linked (Chambers et al. 2012; Chen and Huang 2022; Lee and Lee 2018). Organisms maintain a sophisticated energy allocation between metabolic consumption and the immune system under homeostasis. However, when external or internal stressors, such as infection or starvation, destroy this equilibrium, extra energy is directed to the party with priority demand (Fig. 4) (Bajgar et al. 2015; Banerjee et al. 2019; Kraaijeveld and Godfray 1997; van der Most et al. 2011). To date, the most studied metabolic organs that reveal complex interplay with macrophages in *Drosophila* are fat bodies and muscles.

### Homeostatic condition

During homeostasis, the macrophage population is maintained at a low number with a basal level of energy demand. Surprisingly, the unspecific ablation of blood cells in larvae under normal conditions dramatically reduces adult growth with typical markers of the insulin resistance (P et al. 2020), suggesting a requirement for macrophages in the regulation of physiological processes.

It has been reported that *Drosophila* macrophages generate a PDGF/VEGF-family growth factor, Pvf3, to facilitate lipid storage in the larval fat body (Cox et al. 2021). Both *pvf3* mutant larvae and hemocyte-specific *pvf3* deficient larvae exhibit over 50% reduction in triglyceride content and smaller fat body cell size than in control larvae. The PDGF/VEGF receptor Pvr (also called Vegfr/Stasis) located in fat body cells transduces Pvf signaling to mediate lipid storage in the fat body (Hoch and Soriano 2003; Zheng et al. 2017). Blocking Pvr specifically in the fat body has a very similar effect to that observed in *pvf3* deficient larvae (Cox et al. 2021). It is noteworthy that, PDGFcc, the mouse ortholog of Pvf3, secreted by macrophages, is also essential for lipid storage (Cox et al. 2021).

In healthy *Drosophila* muscles, macrophage-derived Upd3 is required as a metabolic regulator via activating JAK/STAT signaling (Kierdorf et al. 2020). When JAK/STAT signaling is suppressed in larval muscles, reduced activity of AKT, a critical kinase to convey insulin signaling (Roth et al. 2018), and reduced glycogen levels are observed (Yang and Hultmark 2017). Interestingly, another study reported that the loss of the JAK/STAT receptor Domeless in adult muscle tissue leads to hyper-activation of AKT, accompanied by decreased muscle function and a shorter lifespan (Kierdorf et al. 2020). The disparity of these results is possibly due to the different developmental stages investigated. Nevertheless, the macrophage-derived cytokine signal must be received in muscles to regulate AKT-insulin signaling activity and metabolic homeostasis.

### Infection

As described above, upon infection, energy is reallocated to the immune system to support a rapid and efficient response to kill pathogens. The increased number of macrophages secret more Upd3 or adenosine, which systemically activates JAK/STAT signaling and adenosine-associated signaling, respectively, to inhibit metabolic processes (Kim et al. 2013; Mashili et al. 2013; Pean et al. 2017; Woodcock et al. 2015). On the other hand, the immune system also profits from the activities of other tissue/organs. For example, in *Drosophila* larvae infected by parasitoid wasps, sessile macrophage populations are up-regulated and released into the circulation via a fat body-secreted peptide known as Edin (Vanha-Aho et al. 2015).

Similar to the infection situation, blood cells tend to amplify rapidly when a subtype of plasmatocyte is specifically ablated, to maintain a sufficient number of immune cells. In response to this expansion demand, the remaining plasmatocytes produce more Upd3, which activates JAK/STAT signaling and decreases insulin signaling in the fat body, leading to less lipid storage in fat body cells (Shin et al. 2020).

### Nutrient-rich/deficiency condition

In addition to stimulation by pathogenic organisms, the nutrient condition is another critical factor affecting the immune system. Similar to conditions when infected by pathogens, mammalian macrophages also remodel their metabolic strategy to aerobic glycolysis under lipid-rich conditions (Bajgar et al. 2021). In contrast, the metabolic strategy of *Drosophila* macrophages under chronic lipid-rich conditions has yet to be investigated. However, it has been found that a chronic lipid-rich diet in *Drosophila* induces the production of Upd3 by macrophages causing insulin insensitivity and a reduced lifespan via activation of the JAK/STAT pathway (Woodcock et al. 2015). In contrast, under nutrient-deficient conditions, scarce resources need to be reassigned to critical tissues and to support essential processes such as development (Ramond et al. 2020). Meanwhile, the number of macrophages is down-regulated to a basal level to save energy (Dolezal et al. 2019). Fat body-derived adipokine NimB5 binds to macrophages to reduce their proliferation in conditions of nutrient scarcity (Ramond et al. 2020). Blocking NimB5 could result in the production of numerous macrophages, which is likely to deplete energy stores and eventually leads to mortality of larvae raised on a poor diet (Ramond et al. 2020).

## Conclusion and perspectives

*Drosophila* is a powerful model organism to investigate immunity and metabolism due to its comparatively simple immune/hematopoietic or metabolic systems and the conservation of functional mechanisms throughout the evolution (Graham and Pick 2017; Ratheesh et al. 2015; Zhao et al. 2021). In this review, we have discussed the critical role of macrophages in local or systemic metabolism under homeostasis or stress in *Drosophila*. During homeostasis, macrophages are maintained in a basal number and at a basal metabolic level, playing essential roles in metabolism and growth control. However, under stress conditions, for example due to infection or nutrition deficiency, the immune system and other tissues compete for energy and nutrients. When a sudden infection occurs, the number of macrophages is rapidly amplified to defend the host organism against the pathogen (P et al. 2020). In this situation, the immune system has the priority energy demand for organism survival, and metabolism is inevitably affected to save energy resources for the immune system. Likewise, when the nutrition intake is limited, to ensure the survivability of the organism, metabolic processes are primarily supplied and energy demand for macrophages is suppressed. In short, the organism controls the energy flow between the immune system and organic metabolism such that the more important process is prioritized according to the nature of the stress (Fig. 4, Table 2).

**Table 2:** Summary of metabolism alterations in *Drosophila* upon infection or starvation

	Healthy situation	Infection	Poor diet
Number of macrophages	Maintained at a physiological level	Massively increased	Decreased
Energy demand of macrophages	At a basal level	High energy consumption	Lower than a basal/normal level
Metabolic pathways of macrophages	Oxidative phosphorylation	Aerobic glycolysis	
HIF-1α in macrophages	Rapidly degraded	Stabilized	
Upd3 from macrophages	At a constitutive level	Increased	
JAK/STAT signaling in tissues	At a constitutive level	Amplified	
Systemic insulin resistance	OFF	ON	
Interaction between macrophages and fat body	Macrophages generate Pvf3 to facilitate the routine lipid storage in fat body	Adenosine from macrophages inhibits glycogen synthesis in fat body; Edin from fat body promotes the release of sessile macrophages into the circulatory system	Fat body-derived adipokine NimB5 binds to macrophages to reduce their proliferation

Macrophages are highly plastic cells that play multiple roles in organisms. They are also closely associated with many human diseases (Wynn et al. 2013). Morbid obesity, tumors, and infection could cause chronic inflammation induced by continuous macrophage activation and subsequent metabolic disturbances such as insulin resistance (Musselman et al. 2011; Porporato 2016). Indeed, tumors and obese adipose tissues are infiltrated by a large number of macrophages (Geeraerts et al. 2017; Jia et al. 2014; Roth et al. 2018; Weisberg et al. 2003). Tumor-associated macrophages (TAMs) existing in the tumor microenvironment play a pivotal role in supporting tumor growth. The metabolic profile of TAMs is likely biased toward the M2 phenotype (Halbrook et al. 2019). In response to the altered tumor microenvironment, they also present a metabolic switch toward glycolysis to produce altered cytokines and angiogenic factors thereby supporting tumor growth and survival (Puthenveetil and Dubey 2020). Therefore, the regulation of metabolic programs in macrophages could be a therapeutic target to improve the treatment of human diseases.

Macrophages, however, are not the only cell type to perform the phagocytic function. Epithelial follicle cells may serve as phagocytes in the *Drosophila* ovary (Lebo et al. 2021). Glial cells phagocytize apoptotic neuronal cells in the central nervous system (Hilu-Dadia et al. 2018). Moreover, macrophages and glial cells share the same transcription factor Gcm for their development (Trebuchet et al. 2019). It would be interesting to investigate whether these cells undergo the same metabolic shift for performing their phagocytic function.

## Data Availability

This article does not contain any original research data.
